# Atrial fibrillation triggered by pulmonary vein involvement in recurrent lymphoma: successful treatment with pulsed field ablation—a case report

**DOI:** 10.1093/ehjcr/ytaf639

**Published:** 2025-12-11

**Authors:** Ryo Terashima, Yoshinari Enomoto, Risen Hirai, Hisao Hara, Yukio Hiroi

**Affiliations:** Department of Cardiology, National Center for Global Health and Medicine, Japan Institute for Health Security, 1-21-1 Toyama, Shinjuku-ku, Tokyo 162-8655, Japan; Department of Cardiology, National Center for Global Health and Medicine, Japan Institute for Health Security, 1-21-1 Toyama, Shinjuku-ku, Tokyo 162-8655, Japan; Department of Hematology, Tokyo-Kita Medical Center, 4-17-56 Akabanedai, Kita-ku, Tokyo 115-0053, Japan; Department of Cardiology, National Center for Global Health and Medicine, Japan Institute for Health Security, 1-21-1 Toyama, Shinjuku-ku, Tokyo 162-8655, Japan; Department of Cardiology, National Center for Global Health and Medicine, Japan Institute for Health Security, 1-21-1 Toyama, Shinjuku-ku, Tokyo 162-8655, Japan

**Keywords:** Atrial fibrillation, Case report, Intracardiac cytology, Lymphoma, Multimodality imaging, Pulsed field ablation

## Abstract

**Background:**

Atrial fibrillation (AF) is associated with structural and electrical remodelling of the left atrium, but in rare cases, it may occur secondary to external compression or local inflammation. Cardiac involvement of malignant lymphoma is uncommon, and its contribution to AF mechanisms remains poorly understood. We report a case of recurrent lymphoma involving the right inferior pulmonary vein (RIPV), in which pulsed field ablation (PFA) restored sinus rhythm, supported by multimodality imaging and cytological evaluation.

**Case summary:**

A 71-year-old man with a history of follicular lymphoma presented with new-onset AF and palpitations. Contrast-enhanced cardiac computed tomography (CT) revealed an irregular soft tissue lesion involving the RIPV. A retrospective review of a prior non-contrast CT showed subtle thickening in the same region. Pulsed field ablation achieved isolation of all pulmonary veins. Although no abnormal voltage or electrograms were noted near the RIPV, AF terminated immediately upon its electrical isolation. Cytologic analysis of left atrial aspirate obtained near the RIPV revealed Class III atypical lymphoid cells. Post-ablation Positron emission tomography–CT demonstrated intense ^18F-fluorodeoxyglucose (FDG) uptake in the RIPV lesion, supporting metabolically active lymphoma. The patient remained arrhythmia-free without antiarrhythmic therapy at 1-month follow-up and was referred for haematologic treatment.

**Discussion:**

This case illustrates a rare presentation of AF associated with recurrent lymphoma involving the RIPV. Although AF termination after RIPV isolation suggests a pulmonary vein–mediated mechanism, local tumour involvement may have facilitated AF initiation from the RIPV by increasing its arrhythmogenic potential. Multimodal imaging and intracardiac cytology enabled diagnosis without biopsy, while PFA provided safe and effective rhythm control in a structurally compromised region.

Learning points

- Pulsed field ablation allows safe and effective pulmonary vein (PV) isolation in anatomically distorted or tumour-involved regions.
- Multimodality imaging—including contrast-enhanced computed tomography and FDG-Positron emission tomography—combined with left atrial cytology can support the diagnosis of tumour-associated atrial fibrillation (AF).
- Local tumour involvement near the PVs may enhance local arrhythmogenicity and trigger PV-mediated AF, even in the absence of overt electrogram abnormalities.

## Introduction

Atrial fibrillation (AF) is the most common sustained arrhythmia, typically initiated by ectopic activity from the pulmonary veins (PVs) in the setting of atrial remodelling.^1^ While malignancy-associated AF is rare, it can occur when tumours infiltrate the atria or PVs although such cases are rarely documented.^2–5^

Lymphomas rarely involve the heart due to the absence of native lymphoid tissue.^4^ When cardiac involvement occurs, it may cause arrhythmias that are often underrecognized due to subtle clinical manifestations and limited imaging sensitivity.

We report a rare case of recurrent follicular lymphoma involving the right inferior PV (RIPV), in which AF was successfully treated with pulsed field ablation (PFA).

## Summary figure

**Figure:**
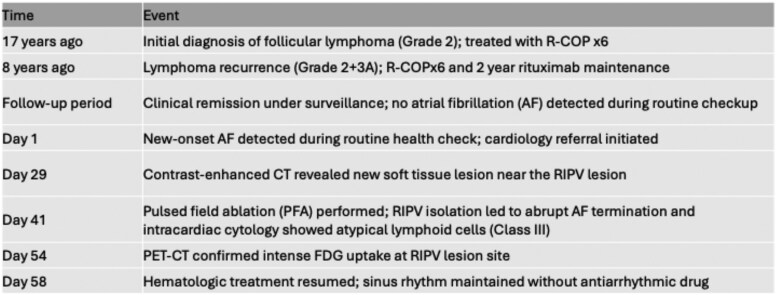


## Case presentation

A 71-year-old man with a history of follicular lymphoma was referred for new-onset AF detected during routine health screening. He reported intermittent palpitations for 3 months but denied chest pain, dyspnoea, or constitutional symptoms such as weight loss or night sweats.

On presentation, vital signs were stable: blood pressure 126/73 mmHg, heart rate irregular at 143 b.p.m., respiratory rate 19 breaths per minute, oxygen saturation 98% on room air, and body temperature 35.6°C. Physical examination revealed an irregularly irregular rhythm without murmurs, gallops, or rales. There was no peripheral oedema, lymphadenopathy, or hepatosplenomegaly.

Laboratory results were unremarkable except for mild renal dysfunction (estimated glomerular filtration rate 49.5 mL/min/1.73 m^2^). Soluble interleukin-2 receptor was within normal limits at 478 U/mL.

**Figure 1:**
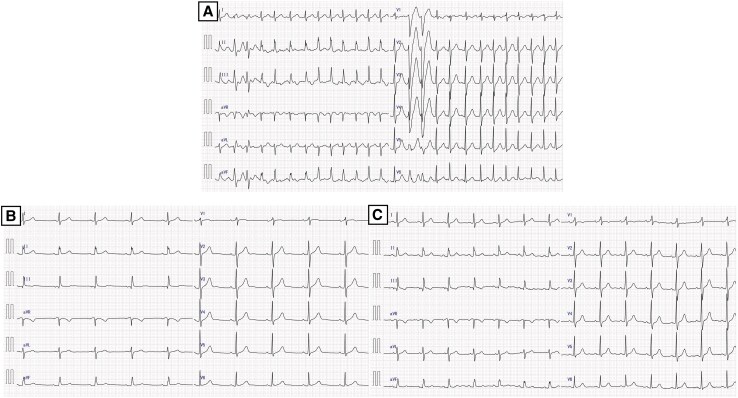
Electrocardiographic findings. (*A*) Electrocardiogram on admission showing atrial fibrillation with a ventricular rate of 143 b.p.m. (*B*) Post-ablation electrocardiogram demonstrating restoration of sinus rhythm. (*C*) One-month follow-up electrocardiogram confirming sustained sinus rhythm.

A transthoracic echocardiogram revealed a left ventricular ejection fraction of 53% without structural abnormalities. Chest radiography was unremarkable. A 12-lead electrocardiogram confirmed AF with a ventricular rate of 143 b.p.m. (*Figure 1A*). Given persistent symptoms and failure of spontaneous cardioversion, catheter ablation was scheduled.

The patient had hypertension, a branch-duct-type intraductal papillary mucinous neoplasm, and recurrent follicular lymphoma. He was initially diagnosed with Grade 2 follicular lymphoma in early 2008 involving the left submandibular lymph node and received six cycles of rituximab, cyclophosphamide, vincristine, and prednisolone (R-COP) chemotherapy over 4 months. A recurrence in late 2017 included a Grade 2 right axillary lymph node and Grade 3A duodenal lesions, treated with six further cycles of R-COP and 2 years of rituximab maintenance. He remained in remission under routine non-contrast computed tomography (CT) and upper endoscopic surveillance.

**Figure 2:**
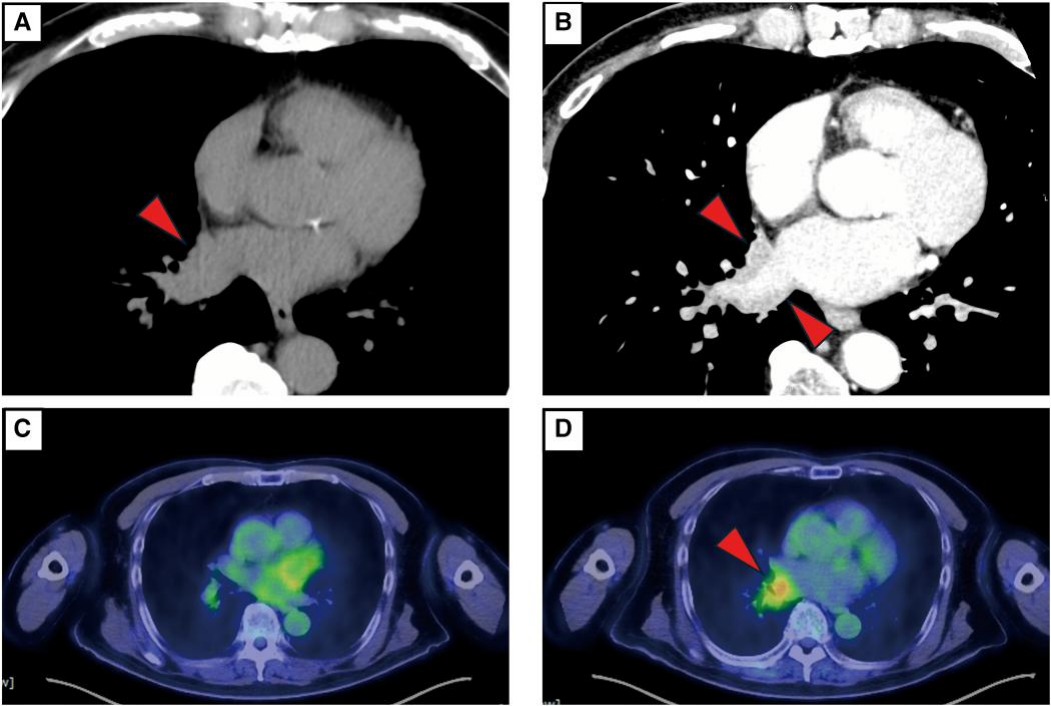
Multimodal imaging findings. (*A*) Retrospective review of follow-up non-contrast computed tomography revealing subtle thickening near the right inferior pulmonary vein. (*B*) Pre-ablation contrast-enhanced computed tomography demonstrating a soft tissue lesion extending into the right inferior pulmonary vein and adjacent left atrial wall, suggestive of lymphoma recurrence. (*C*) Positron emission tomography–computed tomography during remission showing no abnormal FDG uptake in the right inferior pulmonary vein region. (*D*) Post-ablation Positron emission tomography–computed tomography demonstrating intense FDG uptake (SUV 5.11) in the right inferior pulmonary vein region, consistent with metabolically active tumour involvement.

Pre-ablation contrast-enhanced cardiac CT revealed a new irregular soft tissue lesion involving the RIPV, extending towards the posterior left atrial wall and adjacent lung parenchyma (*Figure 2B*). A retrospective review of prior non-contrast CT images revealed subtle thickening in the same region, which had been previously overlooked (*Figure 2A*). These findings raised concern for recurrence of follicular lymphoma.

**Figure 3:**
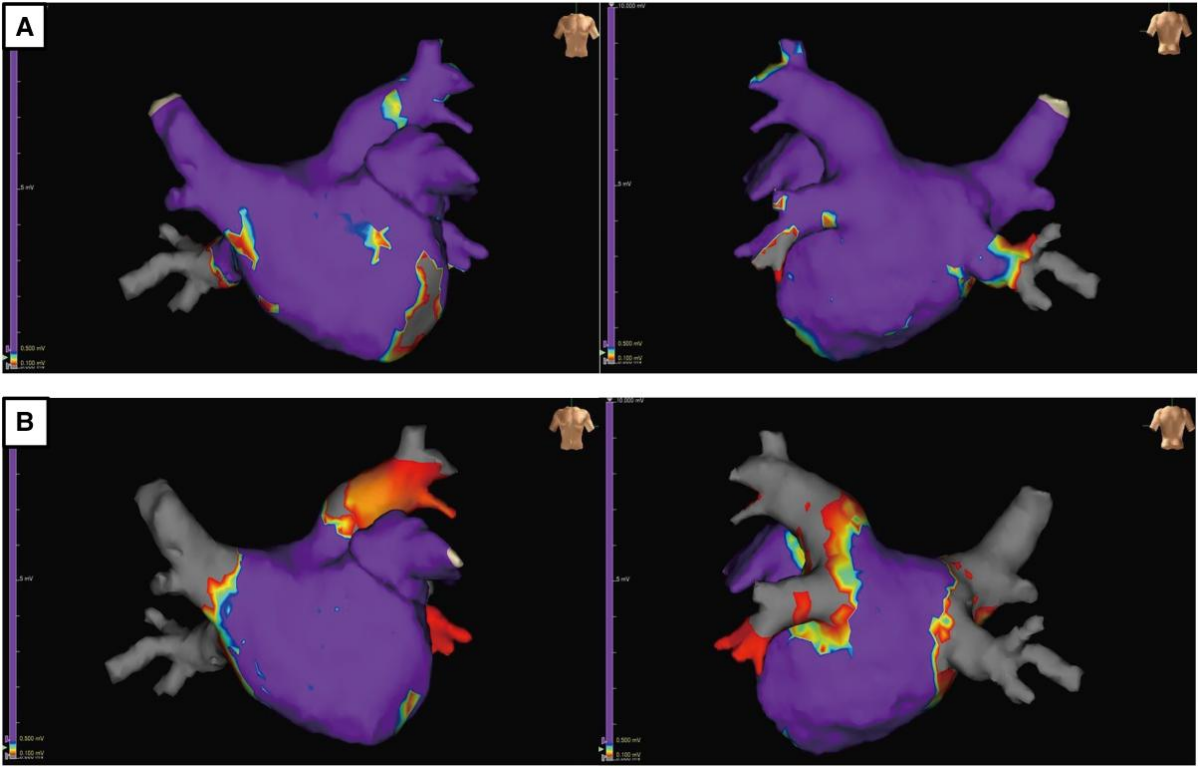
Pre- and post-ablation voltage mapping of the left atrium (left: anteroposterior view; right: posteroanterior view). (*A*) Pre-ablation voltage map showing no abnormal low-voltage areas, including the right inferior pulmonary vein region. (*B*) Post-ablation voltage map confirming complete electrical isolation of all pulmonary veins.

**Figure 4:**
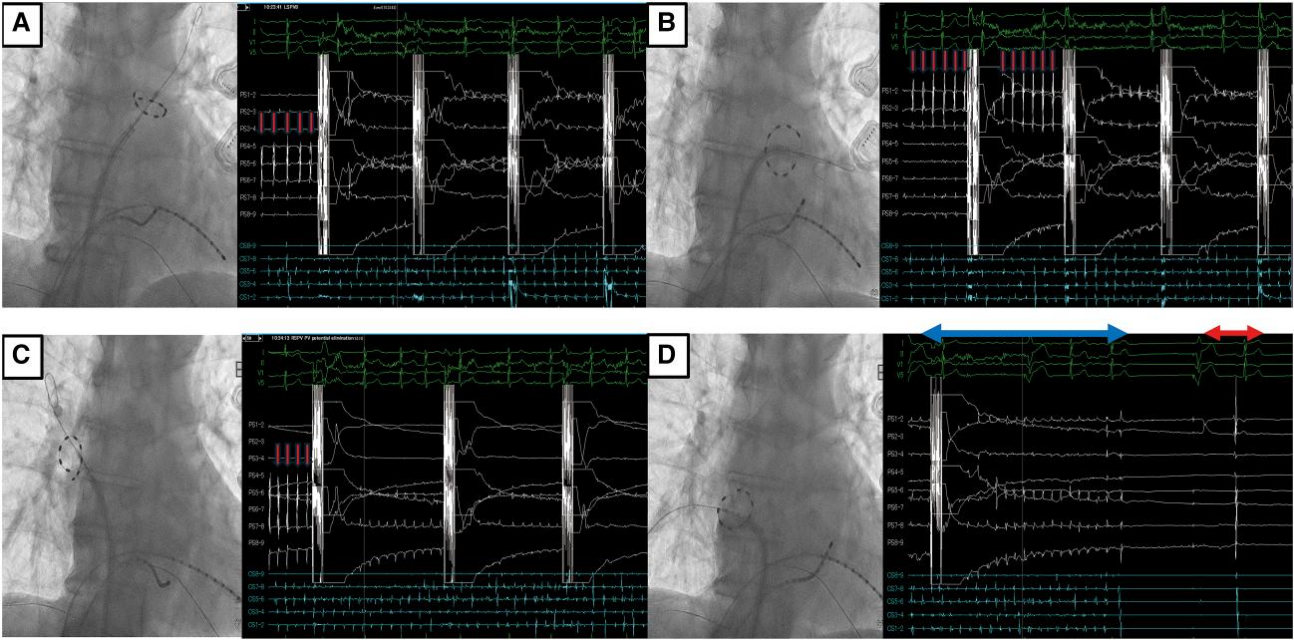
Pulmonary vein isolation using pulsed field ablation. (*A*) The first pulsed field ablation application, targeting the left superior pulmonary vein, eliminated pulmonary vein potentials (red arrows indicate the pulmonary vein potentials prior to ablation). (*B*) The second pulsed field ablation application targeting the left inferior pulmonary vein, terminated residual pulmonary vein potentials. (*C*) Pulsed field ablation targeting the right superior pulmonary vein (RIPV) successfully eliminated pulmonary vein potentials. (*D*) Final pulsed field ablation application to the right inferior pulmonary vein resulted in spontaneous termination of atrial fibrillation and reversion to sinus rhythm after one beat of ventricular pacing (blue arrows indicate atrial fibrillation; red arrows indicate the onset of sinus rhythm).

**Figure 5:**
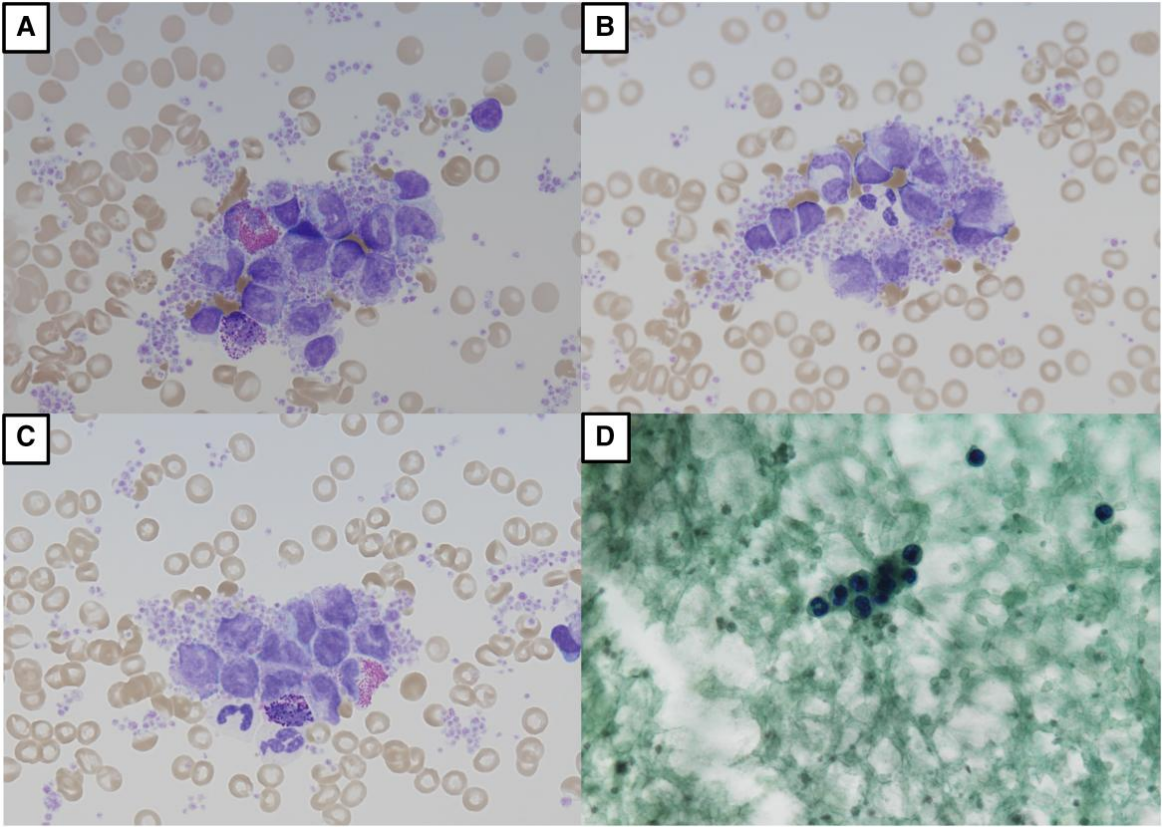
Intracardiac cytologic findings. (*A–C*) Giemsa staining showing clusters of atypical lymphoid cells with enlarged, irregular nuclei and coarse chromatin. (*D*) Papanicolaou staining shows small clusters of atypical lymphoid cells with enlarged, hyperchromatic nuclei and scant cytoplasm, consistent with atypical lymphoid proliferation (Class III cytology).

Catheter ablation was performed using the PulseSelect™ PFA system (Medtronic) in accordance with the manufacturer’s standard ablation protocol. Each PV received eight PFA applications—four within the vein and four at the antrum. When isolation was incomplete, additional applications were administered based on the operator’s judgement. Energy was applied circumferentially at each PV under fluoroscopic and electroanatomic guidance. Voltage mapping (EnSite X system, Abbott) showed no overt low-voltage areas or abnormal electrograms near the RIPV (*Figure 3*). Entrance and exit blocks were confirmed in all veins after sequential PV isolation. Atrial fibrillation persisted despite left and right superior PV isolation but terminated abruptly upon RIPV isolation, suggesting arrhythmogenicity from that region (*Figure 4A–D*). Although lesion depth cannot be directly measured *in vivo*, preclinical studies have demonstrated that PFA produces transmural myocardial lesions ∼3–4 mm in depth, sufficient for durable isolation while minimizing collateral injury.^6^ Left atrial blood was aspirated for cytological evaluation, which revealed Class III atypical lymphoid cells (*Figure 5*). Post-procedural Positron emission tomography (PET)–CT showed focal intense FDG uptake in the RIPV lesion, consistent with metabolically active lymphoma (*Figure 2D*). The patient was referred to haematology and resumed immunochemotherapy.

Edoxaban 60 mg daily was initiated prior to ablation and continued post-procedurally. At 1-month follow-up, the patient remained in sinus rhythm without antiarrhythmic therapy (*Figure 1C*). Anticoagulation is being maintained during the blanking period, with discontinuation to be considered based on rhythm status and chemotherapy-related bleeding risks.

## Discussion

This case illustrates a rare occurrence of AF associated with localized cardiac involvement of recurrent follicular lymphoma. The arrhythmia originated from the RIPV, where a metabolically active lesion was identified. Although AF termination after RIPV isolation suggests a PV-mediated mechanism, local tumour involvement—possibly through compression or early infiltration—might have enhanced the local arrhythmogenicity of the RIPV, facilitating AF initiation.

Tumour-related AF has been described in various cardiac malignancies, with PV or left atrial involvement occasionally provoking atypical AF presentations.^7^ In such cases, structural assessment should complement voltage mapping, as voltage mapping alone may miss anatomically mediated triggers. Although voltage mapping can characterize the atrial substrate, it does not identify the precise triggers of AF. Therefore, in our case, voltage mapping was used as a supplementary tool to confirm the absence of other AF substrates rather than to localize the initiating trigger itself.

Multimodal imaging played a central role in this diagnosis. A retrospective review of a prior non-contrast CT revealed previously overlooked thickening around the RIPV. Contrast-enhanced CT identified a new soft tissue lesion involving the RIPV, prompting the decision to perform intracardiac cytology for further evaluation. Positron emission tomography–CT performed post-ablation subsequently demonstrated intense FDG uptake at the corresponding site, supporting the diagnosis of metabolically active lymphoma.

Cytologic analysis of aspirated left atrial blood revealed Class III atypical lymphoid cells, supporting the diagnosis of secondary cardiac lymphoma. Although rarely used, intracardiac cytology may offer diagnostic value when histologic biopsy is unfeasible due to anatomical constraints or procedural risks.^8^ In our case, the cytology results added confidence to the imaging-based suspicion and facilitated timely haematologic management.

From a procedural standpoint, PFA enabled safe and effective RIPV isolation. In contrast to conventional thermal modalities such as radiofrequency or cryoablation, PFA induces irreversible electroporation to selectively ablate myocardial cells while sparing surrounding tissues, including the oesophagus, phrenic nerve, and vasculature.^9^ Preclinical and clinical studies have demonstrated high tissue selectivity, minimal collateral damage, and a favourable safety profile.^9,10^ These characteristics are especially valuable in anatomically distorted or structurally compromised regions, where conventional thermal ablation carries elevated risk.

To date, comparable cases treated with conventional thermal ablation, such as radiofrequency, have not been reported, likely due to safety concerns in tumour-involved regions. Although the cardiomyocyte selectivity of PFA may theoretically limit its effect on infiltrating tumour tissue, it provided safe and effective rhythm control within a tumour-involved region, while systemic chemotherapy subsequently addressed the underlying malignancy.

In this case, PFA achieved rhythm control without complications, and the patient remained arrhythmia-free without antiarrhythmic therapy at follow-up. Anticoagulation was continued during the blanking period, and long-term management requires individualized decision-making, particularly under active chemotherapy, where bleeding risk and rhythm stability must be carefully balanced.

## Conclusion

To our knowledge, this is the first reported case of AF associated with lymphoma involving the RIPV that was successfully treated with PFA. Local tumour involvement may have enhanced the arrhythmogenicity of the RIPV and triggered AF, even when electrophysiologic findings were unremarkable. Multimodality imaging combined with intracardiac cytology facilitated diagnosis, while PFA provided safe and durable rhythm control in a structurally compromised region. This report underscores the importance of considering malignancy in atypical AF presentations and highlights PFA as a safe and promising therapeutic strategy for rhythm control in anatomically vulnerable, tumour-involved regions.

## Lead author biography


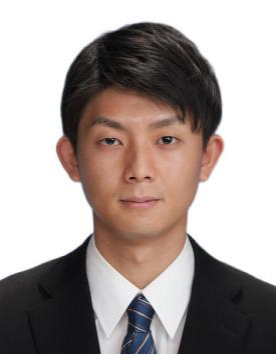


Dr Ryo Terashima, MD, received his medical degree from Yokohama City University School of Medicine. He is currently working as a senior resident cardiologist at the Department of Cardiology, National Center for Global Health and Medicine, Japan Institute for Health Security in Tokyo, Japan.

## Data Availability

The data underlying this article will be shared on reasonable request to the corresponding author.

## Notes

**Consent:** The authors confirm that written consent for submission and publication of this case report, including images and associated text, has been obtained from the patient in line with COPE guidance.
